# Increases in Variation of Barbell Kinematics Are Observed with Increasing Intensity in a Graded Back Squat Test

**DOI:** 10.3390/sports5030051

**Published:** 2017-07-14

**Authors:** Kevin M. Carroll, Kimitake Sato, Caleb D. Bazyler, N. Travis Triplett, Michael H. Stone

**Affiliations:** 1 Department of Sport, Exercise, Recreation, and Kinesiology, East Tennessee State University, 1276 Gilbreath Dr, Johnson City, TN 37614, USA; satok1@etsu.edu (K.S.); bazyler@etsu.edu (C.D.B.); stonem@etsu.edu (M.H.S.); 2 Department of Health and Exercise Science, Appalachian State University, 287 Rivers St., Boone, NC 28608, USA; triplttnt@appstate.edu

**Keywords:** velocity-based training, VBT, resistance training

## Abstract

The purpose of the current study was two-fold: (1) To examine the variation in velocity and power with increasing intensity in the back squat among subjects; and (2) To explore individual subject characteristics as possible explanations for variations of velocity in the back squat. Fourteen recreationally trained male subjects with experience in the back squat agreed to participate in the study (age = 25.0 ± 2.6 years, height = 178.9 ± 8.1 cm, body mass = 88.2 ± 15.8 kg). One-repetition maximums (1RM) were performed for each subject on force platforms with four linear position transducers attached to the barbell. The 1RM assessment was immediately preceded by warm-up sets at 65%, 75%, 85%, and 95% of estimated 1RM for 5, 3, 2, and 1 repetitions, respectively. Mean concentric velocity (MCV) and mean power were recorded for each intensity condition and were analyzed using Pearson correlation to determine the relationship between each variable and relative intensity (%1RM). Statistically significant negative relationships existed between %1RM and MCV (r = −0.892) and mean power (r = −0.604). Between-subject coefficient of variation tended to increase as %1RM increased for both MCV and mean power. These results suggest that MCV is superior to mean power as an indicator of relative intensity in the back squat. Additionally, the between-subject variation observed at higher intensities for MCV and mean power support the use of velocity ranges by strength and conditioning coaches.

## 1. Introduction

Velocity-Based Training (VBT) has recently gained popularity in research and practice [1,2,3,4,5]. VBT is a training strategy emphasizing movement (particularly barbell) velocity for exercise testing and prescription of load in resistance training [6]. Some have suggested fitness qualities (e.g., strength or explosiveness) may be enhanced based on manipulation of barbell velocities during resistance training [1,7]. Moreover, barbell velocities may have a significant impact on the resultant power output of an exercise. This has important implications for performance enhancement, as power output is regarded as a vital factor in performance [8,9], especially for strength-power athletes [10]. However, there is a paucity of research examining both velocity and power output data in the context of VBT application. 

Maximal strength testing, being one of the proposed uses of VBT [6,11,12], merits further examination regarding its usability in practice. Although the topic has been reasonably established [6,11,12], inquiry into specific methodologies and applications is warranted. Maximal strength testing is a widely-used method of establishing one’s strength levels [13,14], prescribing resistance training loads [1,12], and monitoring the adaptive process [15]. One commonly employed method of maximal strength testing in resistance training is the one repetition maximum (1RM). Strong relationships (r > 0.9) have been found between relative intensity (%1RM) and velocity in the free weight back squat, bench press, and deadlift [12,16,17,18]. Furthermore, it has been suggested that coaches may use velocity measurement from submaximal loads to predict 1RM values during training to better inform load selection [6]. This concept requires further investigation to better elucidate how factors such as methodology and individual differences may impact its use. It should be noted that emerging research has questioned the validity of linear 1RM prediction equations, mainly due to the poor reliability of 1RM velocity across multiple trials [19]. 

Although strong relationships have been shown between %1RM and barbell velocity [16], variation in barbell velocity during a 1RM back squat test remains a concern for practical use and should be investigated more thoroughly. Additionally, the meaningfulness of power measures in relation to %1RM is unknown and questionable. There are certainly useful ways to interpret power output, but these remain relatively unknown in comparison to velocity measures. Given that resistance training in the practical setting is rarely performed on Smith machines [15], it would also be worthwhile to examine these relationships utilizing free weights. Therefore, the purpose of the current study was two-fold: (1) To examine the variation in velocity and power with increasing intensity in the back squat among subjects; and (2) To explore individual subject characteristics as possible explanations for variation of velocity in the back squat.

## 2. Materials and Methods

### 2.1. Subjects

Fourteen recreationally trained male subjects with experience in the back squat agreed to participate in the study (age = 25.0 ± 2.6 years, height = 178.9 ± 8.1 cm, body mass = 88.2 ± 15.8 kg). Inclusion criteria for participation included: no current or past injuries affecting the subject’s ability to back squat, experience of at least one year in the back squat, and the ability to perform a parallel back squat. A parallel back squat has been defined as when the hip crease becomes level with the knee joint [20]. Subjects were instructed to avoid any fatiguing activity for 48 h prior to testing and throughout the testing protocol. All subjects read and signed written informed consent documents as approved by the University’s Institutional Review Board.

### 2.2. Experimental Approach

To examine the relationships and variation between both mean concentric velocity (MCV) and mean power with relative intensity in the back squat, kinetic and kinematic variables were collected during a graded 1RM back squat test. The high bar back squat was used for this protocol due to it being a commonly used and important exercise in athlete training programs [15]. A protocol modified from previous research was used to assess back squat 1RM [13], and kinematic variables (velocity and power) were obtained during the testing. 

### 2.3. Methodology

Kinematic variables were collected during a graded 1RM back squat testing protocol for all subjects. All measurements were processed as averages during the concentric portion of each repetition. While peak values are valuable for understanding specific segments of a repetition, average values seem to more accurately represent the concentric portion of each repetition as a whole [6]. Upon arrival to the laboratory, subject height was collected using an electronic stadiometer (Cardinal Scale Manufacturing Co., Webb City, MS, USA), and body mass using a Tanita Body Composition Analyzer BF-350 (Tanita Corporation, Inc., Arlington Heights, IL, USA). During performance testing, force platforms sampling at 1000 Hz (Rice Lake Weighing Systems, Rice Lake, WI, USA) were used to collect kinetic variables. Displacement was collected using four linear position transducers sampling at 1000 Hz (Celesco Measurement Specialties, Chatsworth, CA, USA) attached directly to the barbell (two on each side, see Figure S1) and synchronized with the force platforms using a BNC 2110 connector with an analog-to-digital converter (DAQCard-6063E, National Instruments, Austin, TX, USA) [21]. These four linear position transducers originated at the top of a custom built power rack. A custom LabView program (LabView 8.6, and 2010, National Instruments Co., Austin, TX, USA), which collected both force plate and linear position transducer data, was then used to obtain MCV values during the concentric portion of each lift (Figure S2). Specifically, to calculate velocity data, each of the four transducers provided feedback to the internal rotary encoder as to their respective positions. From there, we were able to calculate both vertical and horizontal displacement of the bar (which all transducers were attached to). Further, using the vertical displacement values, we were able to calculate vertical velocity for MCV using the change in displacement over the change in time.

Prior to the back squat 1RM assessment, and following a dynamic warm-up, all subjects were instructed to perform the concentric portion of each repetition with maximal effort, including the warm-up attempts and all 1RM attempts. The subjects performed the eccentric portion of the squat at a self-selected pace. The investigators chose to allow each subject to use a self-selected pace for the eccentric portion of the squat to maintain ecological validity. Coaches and practitioners who use a velocity-based training approach typically do not standardize kinematics in the practical setting, thus the investigators did not control for kinematics. Subjects were instructed to wait for 1–2 s between repetitions but were permitted to begin each repetition voluntarily. A resistance band was placed at the required depth for each subject as a visual aid for the tester to indicate when the required depth had been reached. The placement of the resistance band was determined by having the subject squat until the qualifications for a parallel squat were met. Upon reaching the required depth for each repetition, a verbal cue was used to instruct each subject to begin the concentric portion of the lift. Verbal encouragement was provided throughout the testing session. 

A modified 1RM protocol was used where subjects performed 65%, 75%, 85%, and 95% of their estimated 1RM for 5, 3, 2, and 1 repetitions, respectively, before attempting their 1RM [13]. After the initial 1RM attempt, subjects continued to increase the load on the barbell by a minimum of 2.0 kg and performed additional 1RM attempts until voluntary failure. Three minutes of rest were given between each warm-up condition and between each 1RM attempt [22]. Each subject achieved their 1RM within 4 attempts. For load conditions containing more than one repetition, average values for the condition were considered for statistical analysis. Repetition-to-repetition reliability for MCV and mean power was assessed using within-subject intraclass correlation coefficients (ICC). For MCV Load 1 (ICC = 0.77), Load 2 (ICC = 0.93), and Load 3 (ICC = 0.88). For mean power Load 1 (ICC = 0.93), Load 2 (ICC = 0.97), and Load 3 (ICC = 0.91). All subsequent load conditions had only one rep, thus reliability was not calculated. The relative intensities used for warm-ups were originally calculated based on the estimated 1RM, but were back-calculated for analysis based on each subject’s achieved 1RM. Kinematic variables were processed using a custom LabView analysis program (LabVIEW, National Instruments, Austin, TX, USA).

### 2.4. Statistical Analysis

Inter-subject MCV and mean power of each load condition, standard deviations (SD), and between-subject coefficient of variation (CV) were calculated for each load condition. To assess the relationship between movement velocity and relative intensity in the back squat, a Pearson product-moment zero-order correlation was performed between the MCV, mean power, and the back-calculated relative intensities for warm-up loads. These data were analyzed using Microsoft Excel^TM^ 2010, (Version 2010, Redmond, WA, USA).

## 3. Results

**Figure 1:**
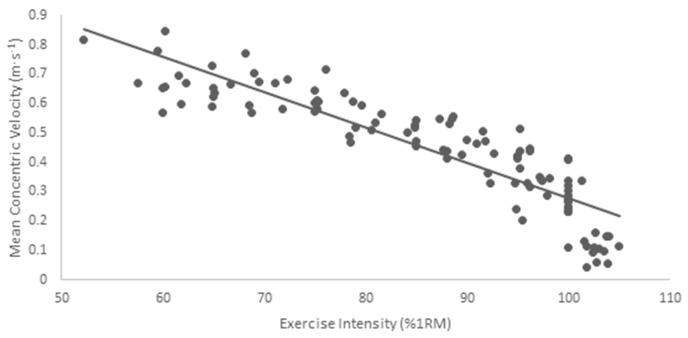
Trend of mean concentric velocity for increasing exercise intensity.

**Figure 2:**
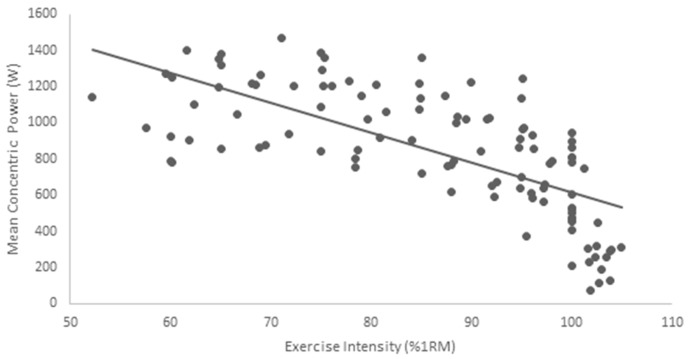
Trend of mean concentric power for increasing exercise intensity.

Both MCV and mean power showed a decreasing trend with increasing load (Table 1 and Table 2). However, between-subject variation of MCV and mean power showed an increasing trend with increasing load. A statistically significant negative relationship (r = −0.892, *p* < 0.001) existed between exercise intensity and MCV (R^2^ = 0.797) (Figure 1). Similarly, a significant negative relationship (r = −0.604, *p* < 0.001) was observed between exercise intensity and mean power (R^2^ = 0.481) (Figure 2).

## 4. Discussion

The current study examined the relationship of MCV and mean power with increasing relative intensity in a graded back squat test. Major findings of this study include: Very large and moderate relationships existed between %1RM and MCV (r = −0.892) or mean power (r = −0.604). Our results support the use of MCV for practitioners wishing to perform strength testing, given the greater relationship between %1RM and MCV compared to mean power. Mean power, while reasonably related to load, was not proven to be a suitable alternative to MCV. Perhaps most important, MCV and mean power between-subject variation increased as relative intensity increased. The coefficient of variation was notably high. This research’s importance comes from the ecological validity both of using free weight exercises that are colloquially used, and of the examination of the efficacy of using velocity- and power-specific recommendations in a group setting. Although a strong relationship exists between both MCV and mean power with %1RM, the increasing variation with increasing intensity suggests that further discussion of the practical efficacy of the protocol is warranted. While we acknowledge that other research has observed small amounts of variation at lower intensities [12], our results support other findings which suggest individual differences may contribute to variation at higher intensities [4].

Our results are in agreement with previous research, which has suggested strong relationships between velocity and %1RM [12,16]. Specifically, our results show that MCV may reasonably equate to %1RM (R^2^ = 0.797). However, mean power showed very modest predictive ability for %1RM (R^2^ = 0.481). This finding suggests that mean power has little impact in the prescription of intensity or load in resistance training. Furthermore, this suggests that MCV is a superior method for the prescription of relative intensity in the free weight back squat compared to mean power. The importance of this finding is evident from the increase in commercially available linear position transducers and other velocity measuring devices being used in recent research [1,17,23,24]. 

A trend of increasing variation in MCV with increasing relative intensity was observed. This finding suggests that as intensity increases, barbell velocity has greater limitations for load prescription. Alternatively, lesser variation existed in MCV at lower intensities. Zourdos et al. observed differences in MCV at higher intensities when comparing more experienced lifters to less experienced lifters, which could in part explain the variability observed in our results. The trend of increasing variability in MCV in the presence of such a strong correlation illustrates the need for closer examination of VBT as it pertains to intensity/load prescription. The authors support the need to establish “velocity ranges” based on observed variation in MCV at specific intensities. It remains to be understood whether these ranges may be individual- or group-specific, and this should be the subject of future research. 

This study was limited by the both the sample size and the population from which subjects were recruited. Recreationally trained males likely do not represent a homogeneous group of athletes of a specific sport. Thus, the differing sport backgrounds of the subjects may have influenced our results. Future research should examine more homogenous groups of athletes and more specifically look at the individual reliability of the velocity-intensity interaction. While the examination of power alongside velocity begins to provide a more complete picture of the movement, future investigations should also consider force outputs and time-specific variables such as impulses or rate of power/force development when examining velocity-based training approaches.

## 5. Conclusions

While a significant strong relationship existed between MCV and relative intensity, increasing variation of both MCV and mean power with a simultaneous increase in intensity in the free weight back squat was observed. This finding suggests that specific velocities are not necessarily related to a specific %1RM in the back squat, but rather that a range of acceptable velocities may exist for a given %1RM. When utilizing a velocity-based approach with athletes, it should be recognized by practitioners that each athlete’s velocity-intensity relationship may be slightly different. This recognition illustrates further that while athlete monitoring based on group averages are important, coaches and sport scientists should examine group variation and individual analyses before making a conclusion.

The finding, in this research, of strong relationships between MCV and %1RM in the free weight back squat agrees with previous research. However, our findings also represent the need to closely examine variations in monitoring data. The trend of increasing variation in MCV with increasing intensity in the back squat suggests that “velocity ranges” should be employed when correlating velocity to a specific %1RM. These findings provide the basis for future research to establish velocity ranges in resistance training practice, further strengthening the emerging field of VBT.

## Figures and Tables

**Table 1:** MCV, SD, and CV associated with increasing load conditions in the back squat.

Condition (Mean %1RM ± SD) *	CV	MCV (m·s^−1^) ± SD	CV
Load 1 (61.89% ± 3.95)	6.39	0.662 ± 0.067	10.116
Load 2 (72.81% ± 6.25)	8.59	0.627 + 0.080	12.690
Load 3 (80.91% + 5.20)	6.43	0.544 + 0.086	15.827
Load 4 (90.61% + 5.83)	6.44	0.478 + 0.079	16.594
1RM Load (100.00% + 0.00)	0.00	0.278 + 0.072	25.773
Failed 1RM Load (102.84% + 1.06)	1.03	0.123 + 0.068	55.119

Note: * %1RM during each load condition was calculated after 1RM testing to determine the mean exercise intensity.

**Table 2:** Mean Power, SD, and CV associated with increasing load conditions in the back squat.

Condition (Mean %1RM ± SD) *	CV	Mean Power (W) ± SD	CV
Load 1 (61.89% ± 3.95)	6.39	1106.8 ± 212.1	19.2
Load 2 (72.81% ± 6.25)	8.59	1128.9 ± 213.3	18.9
Load 3 (80.91% + 5.20)	6.43	1044.3 ± 189.5	18.1
Load 4 (90.61% + 5.83)	6.44	941.1 ± 212.8	22.6
1RM Load (100.00% + 0.00)	0.00	618.2 ± 208.5	33.7
Failed 1RM Load (102.84% + 1.06)	1.03	262.9 ± 169.3	64.4

Note: * %1RM during each load condition was calculated after 1RM testing to determine the mean exercise intensity.

## Supplementary Materials

The following are available online at www.mdpi.com/2075-4663/5/3/51/s1, Figure S1: Set-up configuration of linear position transducers for data collection; Figure S2: LabView collection of vertical displacement-time and vertical force-time data.
