# Supplementary material for: Increases in Variation of Barbell Kinematics Are Observed with Increasing Intensity in a Graded Back Squat Test
Source: Sports (Basel). 2017 Jul 14;5(3):51. doi: 10.3390/sports5030051 (PMC5968951; doi:10.3390/sports5030051)
Supplement: Supplementary file 1 [file sports-05-00051-s001.pdf]

Supplementary Materials: Increases in Variation of Barbell Kinematics Are Observed with Increasing Intensity in a Graded Back Squat Test

Kevin M. Carroll [1,*], Kimitake Sato [1], Caleb D. Bazyler [1], N. Travis Triplett [2] and Michael H. Stone [1]

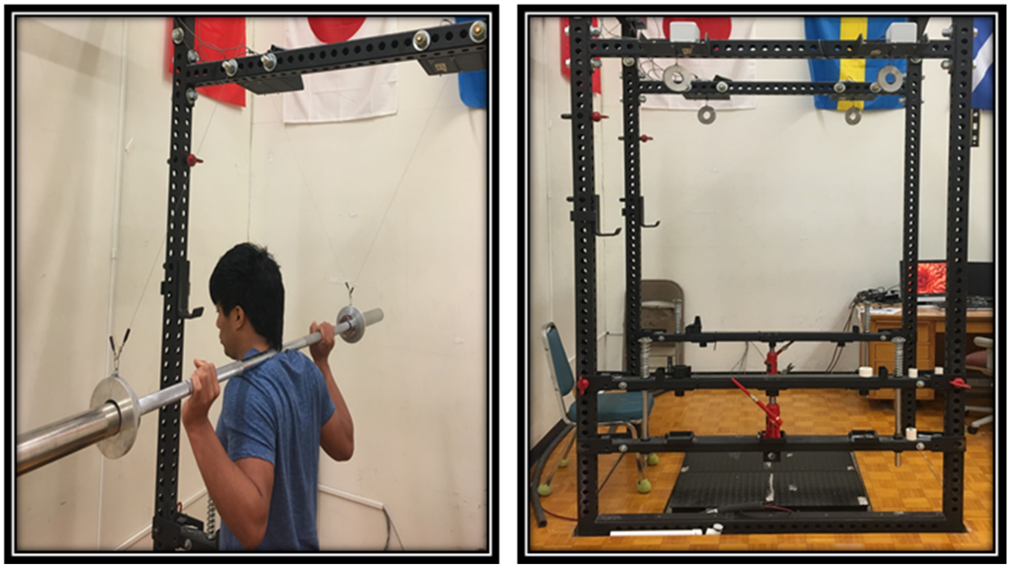

Figure S1. Set-up configuration of linear position transducers for data collection.

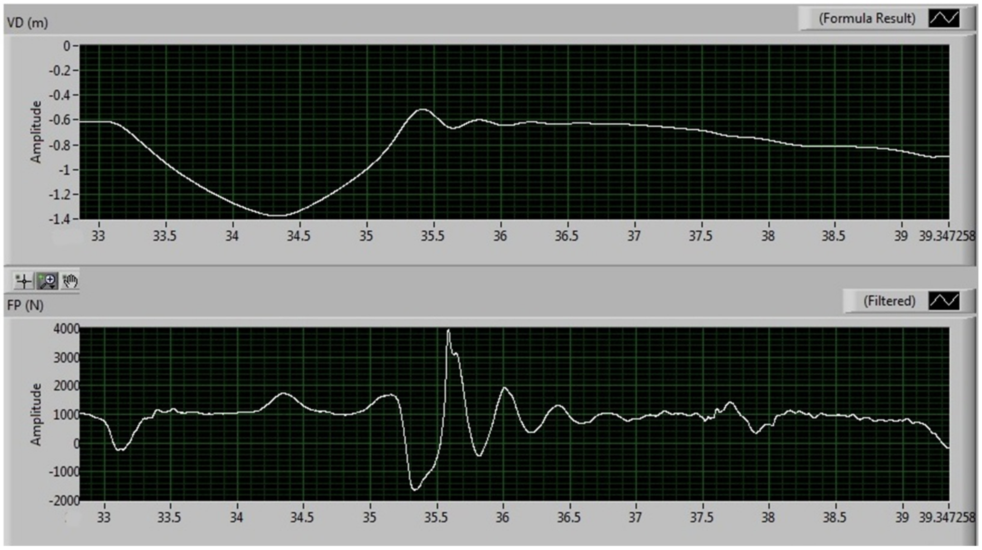

Figure S2. LabView collection of vertical displacement-time and vertical force-time data.
